# Genome-Wide Identification, Phylogenetic Analysis, and Expression Pattern of Polyamine Biosynthesis Gene Family in Pepper

**DOI:** 10.3390/ijms26178208

**Published:** 2025-08-24

**Authors:** Duo Lin, Xianqi Zhao, Qingshan Hu, Su Wang, Yan Zhang, Zijian Xu

**Affiliations:** College of Horticulture, Qingdao Agricultural University, Qingdao 266109, China; linduo73@163.com (D.L.); zhaoxianqi516@163.com (X.Z.); huqingshan12@163.com (Q.H.); wangsu64@163.com (S.W.); zy17604680824@163.com (Y.Z.)

**Keywords:** pepper, polyamines, genome-wide, gene expression, abiotic stress, phytohormone

## Abstract

Polyamines (PAs), including putrescine, spermidine, spermine, and thermospermine, play essential roles in plant growth, development, and responses to stress. However, the structure and function of PA biosynthetic genes in pepper remain poorly characterized. This study aimed to identify PA biosynthesis genes in the pepper genome using bioinformatics approaches and to assess their expression under various stress conditions. A total of 16 PA biosynthesis-related genes were identified, representing members of the arginine decarboxylase (ADC), ornithine decarboxylase (ODC), agmatine iminohydrolase (AIH), N-carbamoylputrescine amidohydrolase (CPA), S-adenosylmethionine decarboxylase (SAMDC), spermidine synthase (SPDS), spermine synthase (SPMS), and ACAULIS5 (ACL5) gene families. These genes encode proteins with an average molecular weight of approximately 40 kDa, primarily localized in the mitochondria and cytoplasm. Promoter analysis revealed multiple *cis*-acting elements associated with stress and phytohormone responsiveness. Gene expression was induced by various abiotic stresses, including saline-alkaline, drought, heat, cold, and hydrogen peroxide, as well as by phytohormones such as abscisic acid, ethylene, salicylic acid, auxin, and gibberellin. Overall, this study provides a comprehensive analysis of PA biosynthesis genes in pepper and highlights their potential roles in stress adaptation and hormone signalling, offering a foundation for further exploration of PA-mediated stress tolerance mechanisms.

## 1. Introduction

Polyamines (PAs) are a class of strongly biologically active, low molecular weight, aliphatic nitrogenous bases produced during biological metabolism. They were among the first key substances discovered in biochemical research [1]. PAs exhibit polycationic properties, typically enabling interactions with polyanionic substances, including nucleic acids, proteins, membrane phospholipids, and pectic polysaccharides, through hydrogen and ionic bonds. These interactions regulate cellular ion balance [2]. Furthermore, PAs enhance the structural stability of proteins, RNA, and DNA, reducing membrane-lipid phase transitions and maintaining membrane integrity [3]. Recent studies have shown that PAs also perform hormone-like functions, such as participating in signal transduction within plant cells and activating downstream responses by directly serving as “second messengers” or indirectly modulating the Ca^2+^ signalling system [4].

In higher plants, several types of PAs are present, with putrescine (Put), spermidine (Spd), and spermine (Spm) being the predominant forms. Additionally, small amounts of cadaverine, thermospermine (Tspm), norspermine (Nspm), and norspermidine (Nspd) have been identified [5]. There are three principal forms of PAs in plants: free, soluble conjugated, and insoluble bound. The accumulation of these forms is essential for plant responses to stress. However, the content and function of PAs are mainly regulated by the dynamics of their synthesis and catabolism [6]. The biosynthesis of PAs in plants primarily proceeds via the arginine (Arg) and ornithine (Orn) pathways. In one pathway, Arg is catalyzed by arginine decarboxylase (ADC) to form agmatine (Agm), which is further converted to Put through the sequential action of agmatine iminohydrolase (AIH) and N-carbamoylputrescine amidohydrolase (CPA). Alternatively, Orn generates Put via ornithine decarboxylase (ODC). The subsequent synthesis of Spd and Spm from Put requires aminopropyl groups donated by decarboxylated S-adenosylmethionine (dcSAM), produced through the action of S-adenosylmethionine synthase (SAM) and S-adenosylmethionine decarboxylase (SAMDC). In this process, spermidine synthase (SPDS) and spermine synthase (SPMS) serve as key catalytic enzymes [7]. ACAULIS5 (ACL5) encodes Tspm synthase, which catalyzes the synthesis of less abundant PAs, including Tspm and Nspm [8]. The accumulation of PAs also depends on catabolic pathways, primarily mediated by copper amine oxidase (CuAO) and polyamine oxidase (PAO). Put is degraded by CuAO, whereas the catabolism of Spd and Spm is regulated by PAO [9].

PAs contribute to various aspects of plant growth and development, including cell division and differentiation, root elongation, flower development, and fruit ripening [10]. Increasing evidence shows that PAs are closely associated with stress responses and regulate plant stress resistance. Early research established that potassium (K^+^) deficiency increases Put accumulation in plants, prompting further investigations into the association between PAs and stress tolerance [11]. Numerous recent studies have shown the role of PAs in plant responses to abiotic stresses, including oxidative, salinity, drought, high temperature, and low temperature stress [12,13]. Stress-induced increases in PA levels are generally accompanied by enhanced activity of PA biosynthetic enzymes and upregulation of biosynthetic genes [14]. For example, expression of *ADC* and *ODC* genes is associated with drought and salinity tolerance, with ADC activity elevated under salinity stress [15,16]. In rice seedlings, salinity and drought stress induce the expression of *SAMDC1* [17]. In tea plant, the *CsSPMS* gene is rapidly upregulated under cold stress [18]. However, the dynamics of PA levels differ across plant species and stress conditions. For example, levels of Put, Spd, and Spm increase markedly during low temperature acclimation in citrus [19], whereas many plants exposed to salinity stress show decreased Put and increased Spd and Spm contents; the (Spd + Spm)/Put ratio rises with increasing salinity, thereby enhancing salt tolerance [20]. Although substantial evidence supports an association between PAs and plant stress responses, the differential regulation of PA biosynthesis pathways under various stresses remains largely unknown across plant species.

Pepper (*Capsicum annuum* L.) is a major crop in the Solanaceae family, originating in Central and South America and now widely cultivated worldwide. As a warm-season crop with shallow root systems, pepper is frequently subjected to abiotic stresses, including drought, salinity, and low temperature. These stresses hinder growth and development, and may even result in plant death. Previous multi-omics analyses identified six *CaPAO* genes in pepper, with *CaPAO2* and *CaPAO4* positively contributing to cold stress responses [21]. In addition, the *CaSPDS* gene, which participates in PA biosynthesis, was also shown to respond to cold stress [22]. However, comprehensive studies on other PA biosynthetic genes in pepper are lacking, particularly regarding their response to different stress conditions. Therefore, this study utilized the T2T pepper genome database to identify candidate PA biosynthetic genes, analyze their gene structures, promoter *cis*-elements, protein properties, chromosomal localization, and phylogenetic relationships. The expression patterns in various tissues, organs, and under different hormonal and abiotic stresses were also examined to provide a basis for elucidating PA function and its role in stress resistance in pepper.

## 2. Results

### 2.1. Identification of PA Biosynthesis Genes in Pepper

**Figure 1:**
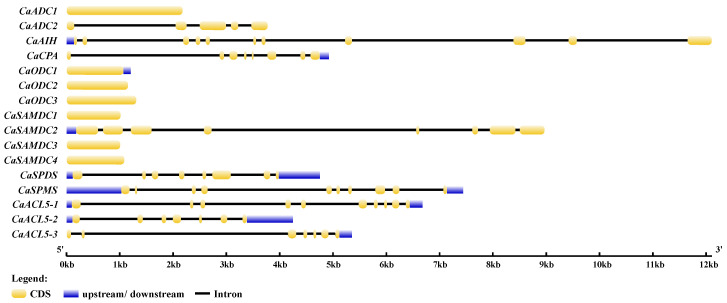
Gene structure of the pepper polyamine biosynthesis genes mapped using GSDS website. The yellow blocks indicate the coding sequence (CDS), the blue blocks represent the region upstream or downstream of the genes, and the black lines indicate the introns. The lengths of the DNA sequences are indicated by the scale bar.

PA biosynthetic genes in pepper (*Capsicum annuum* L.) were systematically identified using the HMMER model (Table 1). Two *ADC* genes (*CaADC1* and *SlADC2*) and three *ODC* genes (*CaODC1*, *CaODC2*, and *CaODC3*) were detected in the telomere-to-telomere gapless genome. Among these, only *CaADC2* contained introns in its genomic sequence (Figure 1). Two further genes responsible for Put biosynthesis, agmatine iminohydrolase (*CaAIH*, CaT2T12g01633) and N-carbamoylputrescine amidohydrolase (*CaCPA*, CaT2T11g00431), were also identified; CaAIH and CaCPA contained 10 and 7 introns, respectively (Figure 1).

In the Spd, Spm, and TSpm biosynthetic pathways, one spermidine synthase (*CaSPDS*), one spermine synthase (*CaSPMS*), and three *ACL5* genes (*CaACL5-1*, *CaACL5-2*, and *CaACL5-3*) were identified. All these genes exhibited multiple introns, with *CaSPMS* and *CaACL5-1* each containing 9 introns, and the remaining *CaACL5* genes containing 6 introns. In addition, four *SAMDC* genes (*CaSAMDC1*, *CaSAMDC2*, *CaSAMDC3*, and *CaSAMDC4*) were identified, with only *CaSAMDC2* containing 7 introns (Figure 1).

The physicochemical properties of the encoded proteins were analyzed using ProtParam. The results indicated that these proteins had an average sequence length of approximately 400 amino acids and a mean molecular weight of approximately 45 kDa (Table 2). CaAIH, CaCPA, CaSAMDC4, and three CaACL5 proteins were predicted to be unstable based on their instability indices (Table 2).

### 2.2. Cis-Acting Elements in the Promoters of PA Biosynthesis Genes in Pepper

**Figure 2:**
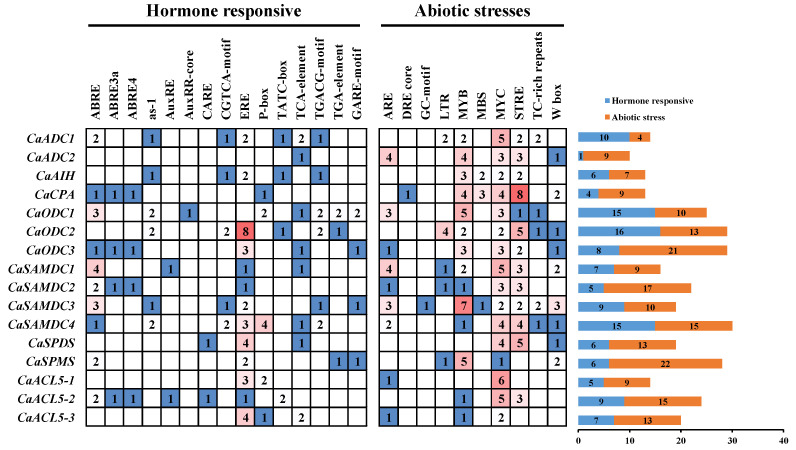
Analysis of *cis*-acting elements in the promoter of polyamine biosynthesis genes. The promoter sequences were identified as 2000 bp upstream of ATG to analyses *cis*-acting elements. The gradient colors in the grid represent the number of *cis*-acting elements in the promoter of polyamine synthesis genes. The multicolor histogram indicates the number of different categories of *cis*-acting elements in these genes.

*Cis*-acting elements play a critical role in transcriptional regulation through interactions with trans-regulatory factors [23]. To clarify the regulatory potential, 2000 bp promoter sequences of PA biosynthetic genes were extracted and analyzed for *cis*-acting elements. A total of 57 *cis*-acting elements were identified. Universal elements such as the TATA-box and CAAT-box were found in all promoters, whereas the remaining 55 *cis*-acting elements were classified into four categories: plant growth and development (9), light-responsive (19), hormone-responsive (15), and abiotic/biotic stress-related (12) (Table S2). To further evaluate the role of *cis*-acting elements under stress and hormonal treatments, the number of hormone- and stress-responsive elements in each promoter was determined (Figure 2). The 15 hormone-responsive elements included motifs related to ABA, jasmonic acid (JA), SA, GA, auxin, and ethylene signalling, such as AREB, as-1, TGA-element, GARE-motif, TCA-element, TGACG-motif, and ERE. The 12 stress-related elements included *cis*-acting elements that respond to low temperature, drought, and dehydration stresses, as well as elements that interacting with stress-related transcription factor, such as MYB, MYC, and W-box.

### 2.3. Phylogenetic Analysis of PA Biosynthesis Genes

**Figure 3:**
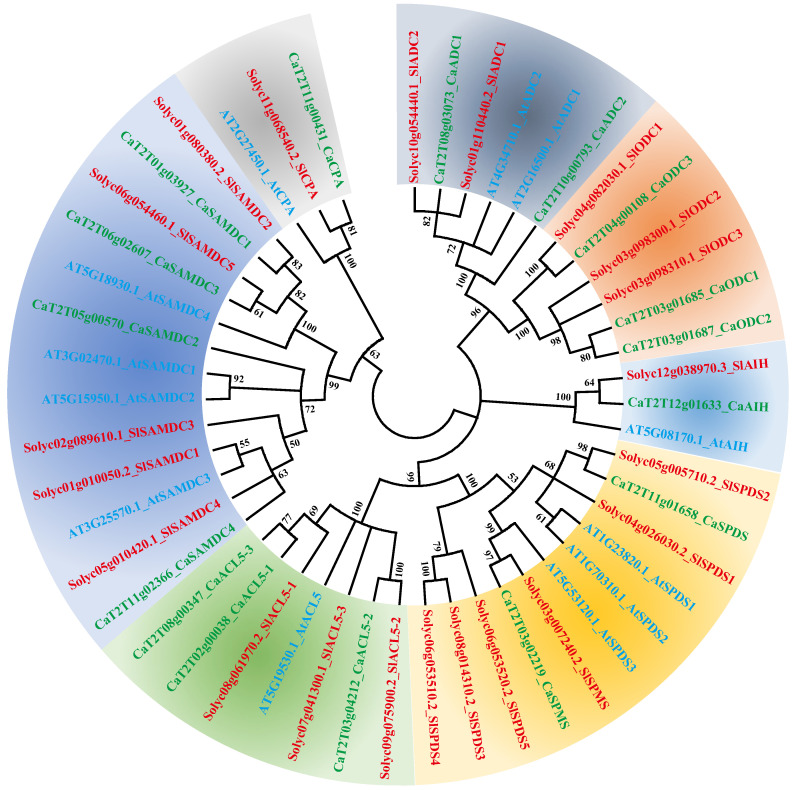
Phylogenetic analysis of polyamine biosynthesis proteins. The protein sequences of polyamine biosynthesis were extracted from pepper (Ca, green font), tomato (Sl, red font) and *Arabidopsis* (At, blue font). Multiple sequence alignments were generated with the *ClustalW*, and the phylogenetic tree was constructed using neighbor-joining method (NJ). ADC, arginine decarboxylase; AIH, agmatine iminohydrolase; CPA, N-carbamoylputrescine amidohydrolase; ODC, ornithine decarboxylase; SAMDC, S-adenosylmethionine decarboxylase; SPDS, spermidine synthase; SPMS, spermine synthase; ACL5, ACAULIS5.

To assess the evolutionary relationships among PA biosynthetic genes, protein sequences from *Arabidopsis*, tomato, and pepper were used to construct a phylogenetic tree. The analysis grouped PA biosynthetic proteins into seven major clades. The ODC group consisted of six members, and three from pepper and three from tomato—whereas *Arabidopsis* lacked ODC genes in its genome. Notably, SPDS and SPMS proteins clustered within the same clade (Figure 3). Pepper and tomato, both members of Solanaceae, showed close evolutionary proximity, resulting in their homologous proteins clustering together, in contrast to those from *Arabidopsis*. Alignment analysis further demonstrated that PA biosynthetic proteins from pepper possessed conserved regions similar to those in tomato and *Arabidopsis* (Figures S1–S8).

### 2.4. Chromosomal Localization and Duplication of PA Biosynthesis Genes in Pepper

**Figure 4:**
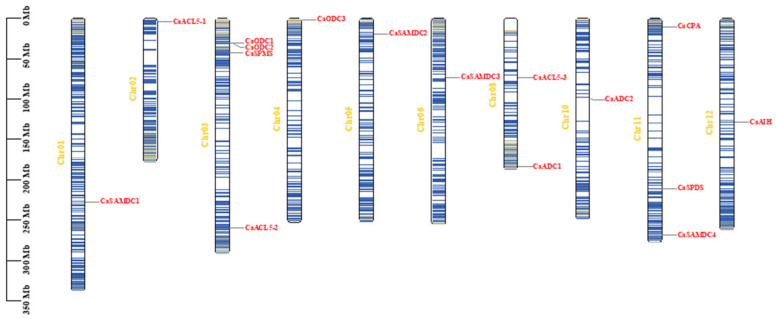
Chromosomal locations of polyamine biosynthesis genes. Chromosomal mapping was based on the physical position in 12 pepper chromosomes. The polyamine biosynthesis genes marked in red. The yellow numbers represent chromosome numbers. Blue to red on the chromosome indicates gene density. The scale on the left is in megabytes (Mb).

Sixteen PA biosynthetic genes were mapped to ten chromosomes in the pepper genome, with only chromosomes 07 and 09 lacked target genes. The gene locations were as follows: *CaSAMDC1* (Chr01), *CaACL5-1* (Chr02), *CaODC3* (Chr04), *CaSAMDC2* (Chr05), *CaSAMDC3* (Chr06), *CaADC2* (Chr10), *CaAIH* (Chr12), *CaACL5-3* and *CaADC1* (Chr08), *CaCPA*, *CaSPDS*, and *CaSAMDC4* (Chr11), and *CaODC1*, *CaODC2*, *CaSPMS*, and *CaACL5-2* (Chr03) (Figure 4).

**Figure 5:**
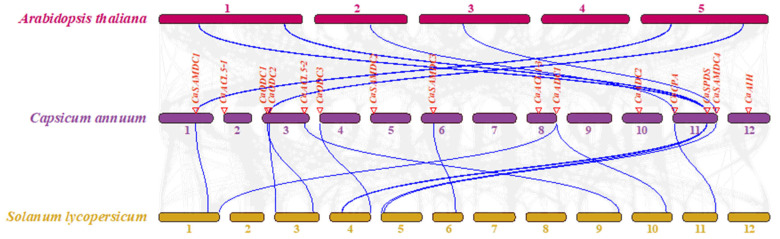
Collinear analysis of polyamine biosynthesis genes among pepper (*Capsicum annuun*), tomato (*Solanum lycopersicum*) and Arabidopsis (*Arabidopsis thaliana*). The segmental duplicated genes are connected by blue lines in different species. Chromosome numbers are located above or below the chromosomes.

Collinearity analysis was performed to assess gene duplication events between pepper and two model species, tomato and *Arabidopsis*. Six pairs of PA biosynthetic genes were identified as duplicated between the pepper and *Arabidopsis* genomes, whilst twelve pairs were identified between pepper and tomato. Genes including *CaSAMDC1*, *CaODC2*, *CaCPA*, *CaSPDS*, and *CaSAMDC4* in pepper were homologous in both model genomes across all duplication events. The *CaSPDS* gene in pepper showed two homologous sites in both *Arabidopsis* and tomato. Among all PA biosynthetic genes, only *CaAIH* was not associated with duplication events in the pepper and tomato genomes (Figure 5).

### 2.5. Organ-Specific Expression Patterns of Pepper PA Biosynthesis Genes

**Figure 6:**
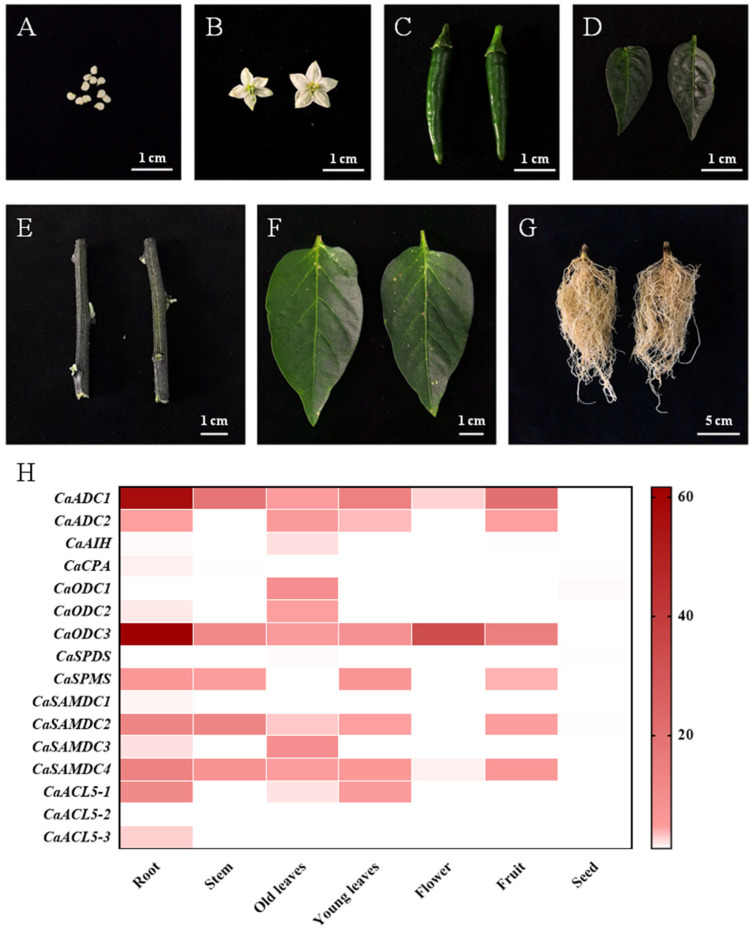
The different tissues and organs of pepper, as well as the expression profiles of polyamine biosynthesis genes. (**A**–**G**) The different tissues and organs of ‘Ruila Jiaozhongyu’ pepper are (**A**) seeds, (**B**) flowers, (**C**) fruits, (**D**) young leaves, (**E**) stems, (**F**) mature leaves, and (**G**) roots. (**H**) The heatmap indicates the expression patterns of polyamine biosynthesis genes in various tissues and organs of peppers. *CaACTIN* was used as an internal control and the relative expression levels of genes were based on the gene expression of seed. Values represent the averages of three independent measurements.

The specific expression patterns of genes provide insights into their functional roles in plant growth and development [24]. In this study, the organ-specific expression profiles of PA biosynthetic genes were assessed using RT-qPCR in pepper. The expression of PA biosynthetic gene in seeds was used as a reference to calculate relative expression levels across different tissues and organs. The results showed that CaADC1, CaODC3, and CaSAMDC4 genes displayed high expression in various tissues and organs (Figure 6H). Comparatively, CaADC1, CaODC3, CaCPA, CaSPMS, CaSAMDC1, CaSAMDC2, CaSAMDC4, CaACL5-1, and CaACL5-3 exhibited the highest expression in roots, whereas CaADC2, CaAIH, CaODC1, CaODC2, and CaSAMDC3 were predominantly expressed in mature leaves. Notably, CaSPDS and CaACL5-2 did not show significant expression in any of the examined tissues or organs.

### 2.6. Effect of Exogenous Phytohormones on PA Biosynthesis Gene Expression

**Figure 7:**
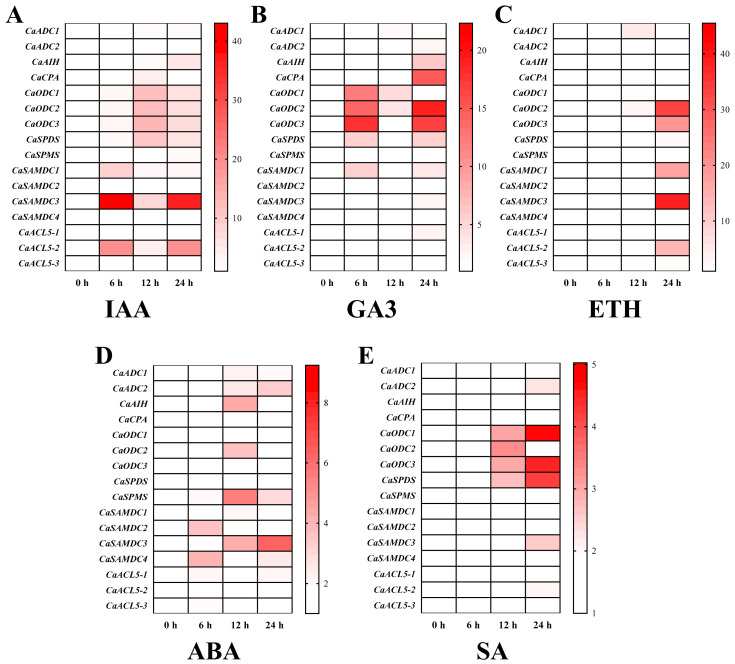
The expression profiles of pepper polyamine biosynthesis genes by exogenous phytohormones. (**A**–**E**) represent the expression of polyamine biosynthesis genes in pepper leaves for six different phytohormones treatments. IAA: 100 μM indole-3-acetic acid; GA3: 100 μM gibberellin; ETH: 100 μM ethylene; ABA: 100 μM abscisic acid; SA: 100 μM salicylic acid. *CaACTIN* was used as an internal control and the relative expression levels of genes were based on 0 h. Values represent the averages of three independent measurements.

Recent research indicates that the association between polyamines and phytohormones enhances plant tolerance to abiotic stress [25]. In this study, several hormones were applied exogenously to the leaves of pepper seedlings, and PA biosynthetic gene expression was measured. The results demonstrated that different hormones induced the expression of PA biosynthetic genes (Figure 7). The most pronounced induction of *CaSAMDC3* expression was observed following auxin treatment, followed by *CaACL5-2*, with peaks at 6 h and 12 h. Additionally, genes in the *CaODC* family, *CaSPDS*, and *CaSAMDC1* were also significantly upregulated (Figure 7A). Expression of the *CaODC* gene family was strongly induced by both gibberellin and salicylic acid treatments. *CaCPA* and *CaAIH* expression was significantly elevated by gibberellin, whereas *CaSPDS* expression was strongly induced by salicylic acid (Figure 7B,E). The expression of *CaODC2*, *CaODC3*, *CaSAMDC1*, *CaSAMDC3*, and *CaACL5-2* was upregulated in response to exogenous ethylene and peaked at 24 h (Figure 7C). Furthermore, *CaSPMS* and the *CaSAMDC* gene family were induced by abscisic acid treatment (Figure 7D).

### 2.7. Differential Expression of PA Biosynthesis Genes During Abiotic Stress

**Figure 8:**
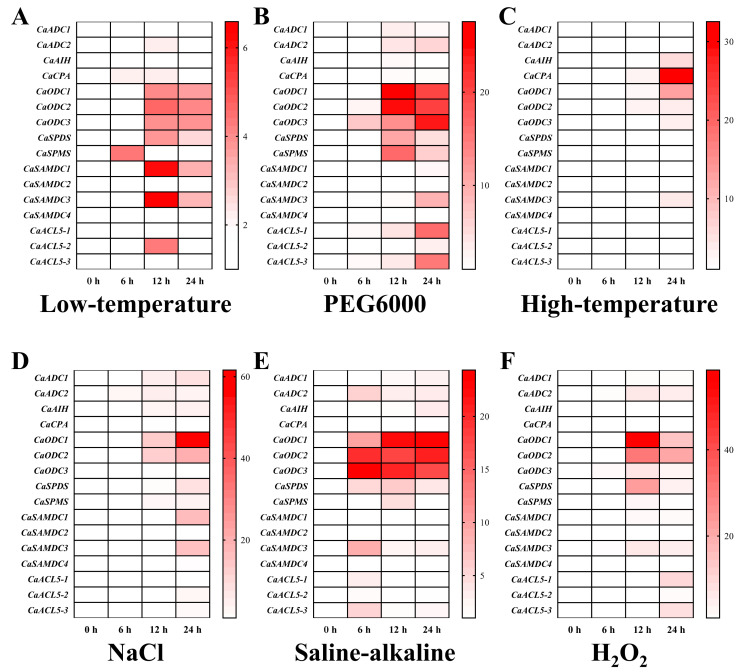
The expression profiles of pepper polyamine biosynthesis genes during abiotic stress. (**A**–**F**) represent the expression of polyamine biosynthesis genes in pepper leaves for six different stress treatments. Low-temperature: 4 °C; PEG600: 20% polyethylene glycol-6000; High-temperature: 42 °C; NaCl: 200 μM NaCl; Saline-alkaline: 200 mM saline-alkaline solution; H_2_O_2_: 100 mM hydrogen peroxide. *CaACTIN* was used as an internal control and the relative expression levels of genes were based on 0 h. Values represent the averages of three independent measurements.

The expression profiles of pepper PA biosynthetic genes under abiotic stress, including low temperature, PEG-6000, high temperature, NaCl, saline–alkaline, and H_2_O_2_ treatments, were investigated. The results showed that most PA biosynthetic genes were induced by low temperature, PEG-6000, NaCl, and saline–alkaline stresses, whereas only a subset was responsive to high temperature and H_2_O_2_ (Figure 8). Differential expression patterns were observed under temperature stress, with *CaODC*, *CaSAMDC3*, and *CaACL5-3* genes induced by both low and high temperatures (Figure 8A,C). Interestingly, the expression of the *CaCPA* gene is induced only by temperature stress. Nearly all PA biosynthetic genes, except *CaSAMDC2*, were upregulated following PEG-6000, NaCl, and saline–alkaline treatments (Figure 8B,D,E). Among them, the three *CaODC* genes and *CaSAMDC3* were induced under all abiotic stress conditions. These findings suggest that polyamines may contribute to the response to abiotic stress in pepper.

## 3. Discussion

PA biosynthetic pathways have been extensively studied in many higher plants, including tomato, sweet orange, and wheat [26,27,28]. In this study, we identified sixteen non-redundant PA biosynthetic genes in the telomere-to-telomere gapless genome of pepper. Although the PA biosynthetic pathway is evolutionarily conserved, the number and types of genes differ across species. For instance, *ODC* genes are absent in *Arabidopsis* [29], but different numbers of *ODC* genes are present in tomato and wheat [26,28]. In pepper, three *ODC* genes were identified; all are intron-free and predicted to be localized in mitochondria (Figure 1 and Table 1). Subcellular localization of biosynthetic proteins provides insight into both their spatial distribution and their functional roles within the cell [30]. However, the actual subcellular compartments of pepper PA biosynthetic proteins have not been experimentally verified, which limits understanding of polyamine function in pepper. In this study, only CaAIH protein was predicted to be extracellular, five proteins from the ADC and ODC families were predicted to be mitochondrial, and the other ten proteins were predicted to be cytoplasmic (Table 1). Comparisons with wheat and *Arabidopsis* showed that SPDS, SPMS, and ACL5-1 were cytoplasmic, whereas SAMDC proteins varied in predicted localization across species [4,26]. Although bioinformatic prediction is informative, experimental confirmation is needed to clarify the precise localization and biological functions of these proteins.

Phylogenetic analysis is an essential approach for elucidating the evolutionary relationships of genes across species [31]. Here, we compared the protein sequences of PA biosynthetic genes from pepper, *Arabidopsis*, and tomato. The phylogenetic tree grouped the proteins into seven clusters, with functionally equivalent proteins from different species clustering together (Figure 3). SPDS and SPMS proteins clustered in the same branch, consistent with their roles in catalyzing aminopropyl transfer to form Spd and Spm [32,33]. Their shared conserved domains likely underlie their inability to be resolved into separate branches in phylogenetic analysis. Although clustering of proteins with similar functions is apparent, the evolutionary diversification of PA biosynthetic gene families in pepper warrants further study. It is well established that gene and genome duplication events play a key role in the emergence of new gene functions and in the diversification of genetic systems [34,35]. Comparative syntenic mapping provides insight into the evolutionary history of gene families [36]. Our comparative analysis revealed that most PA biosynthetic genes in pepper are more closely related to those in tomato than to those in *Arabidopsis* (Figure 5), reflecting the shared evolutionary background of Solanaceae crops. However, none of the *CaAIH* or *CaACL5* genes in pepper were syntenic with genes in tomato or *Arabidopsis*, suggesting a higher degree of evolutionary divergence in these gene families.

Transcriptional regulation of plant genes depends on interactions among *cis*-acting elements and *trans*-acting factors, most of which are located in the promoter region upstream of the gene. The promoter regulates gene transcription by facilitating precise binding of RNA polymerase, thereby ensuring accurate initiation of transcription [37]. Many studies have established the importance of PAs in regulating plant stress tolerance [25]. In this study, we analyzed the promoter regions of pepper PA biosynthetic genes and identified numerous *cis*-acting elements associated with abiotic stress and phytohormone response (Figure 2 and Table S2). Notably, all promoters contained MYC recognition sites—conserved *cis*-acting elements bound by MYC transcription factors—which are central mediators of plant responses to diverse stresses [38]. Additional elements, including MYB recognition sites and STRE motifs, were also widely present. MYB elements are recognized by MYB transcription factors, which play crucial roles in stress-responsive gene regulation in plants [39]. In trifoliate orange (*Poncirus trifoliata* L. Raf.), the MYB *cis*-element in the PtADC promoter of trifoliate orange interacts with PtsrMYB to enhance dehydration tolerance [40]. The STRE motif, originally identified as a binding site for the Msn2p/Msn4p activator in yeast [41], is also found in wheat PA gene promoters [26], although plant-specific binding proteins have yet to be identified. The promoter of pepper PA biosynthetic genes also contains phytohormone-responsive elements, including ABRE (ABA response), ERE (ethylene response), TGACG- and CGTCA-motifs (jasmonate response), and TCA-element (salicylic acid response). ABRE, in particular, is a key *cis*-element in ABA signalling and interacts with AREB/ABF transcription factors. Disruption of these factors, as in *areb1*/*areb2*/*abf3* triple mutants, leads to reduced expression of stress-responsive genes and impaired dehydration tolerance [42]. In trifoliate orange, PtrABF2 directly binds the ABRE motif in the *PtrADC* promoter and enhances drought tolerance through increased Put accumulation [43]. The presence of multiple hormone-responsive elements indicates that PAs are integrated into broader hormone signalling networks. These findings suggest that the expression of pepper PA biosynthetic genes is coordinately regulated by abiotic stress and phytohormones.

Under adverse conditions, plants have evolved diverse adaptive strategies to ensure survival. Among these, the accumulation of PAs plays a key role in counteracting stress. Endogenous PAs, including Put, Spd, and Spm, are known to accumulate to varying degrees under stress, and their function is often integrated with phytohormone signalling pathways in response to environmental stressors [44,45]. The expression of PA biosynthetic genes is dynamically regulated by abiotic stresses and phytohormone signals, supporting plant adaptation through transcriptional control [42,46]. In *Arabidopsis*, twelve genes encode enzymes responsible for PA biosynthesis, including *AtADC1/2*, *AtSAMDC1/2*, *AtSPDS1/2*, *AtSPMS*, and *AtACL5*, all of which are strongly induced by at least one abiotic stress such as salt, drought, or cold [47]. Loss-of-function mutants for *atadc1* and *atadc2* exhibit reduced salt and cold tolerance [48], whereas the *atspms/atacl5* double mutant displays diminished tolerance to salt and drought [49]. Similarly, in apple, *SAMDC1* and *SAMDC2* are mainly induced by salt and temperature stress [50], and in cucumber, *SAMDC3* is upregulated by salt stress [51]. In pepper, *CaSPDS* is rapidly induced under low-temperature stress, and overexpression of *CaSPDS* in *Arabidopsis* enhances cold tolerance, antioxidant enzyme activities, Spd content, and the expression of cold-responsive genes [22]. In this study, low-temperature stress significantly upregulated not only *CaSPDS* but also *CaODC1/2/3*, *CaSPMS*, *CaSAMDC1/3*, and *CaACL5-2*. Reports have indicated that polyamine biosynthesis in plants primarily occurs through the arginine pathway, in which ADC, AIH, and CPA play important roles [29]. However, various abiotic stresses induce the expression of *ODC* genes in pepper, suggesting that the pepper PA biosynthesis genes are induced stress via the ornithine pathway. Moreover, the *CaODC* genes appear to be highly sensitive to osmotic stress, as they are significantly induced under PEG-6000, NaCl and saline-alkaline stress, which all share the common effect of altering the cellular osmotic potential. Similarly, the *CaCPA* gene is more sensitive to temperature stress and only induced by high- or low-temperature stress (Figure 8). Additional PA biosynthetic genes in pepper were induced by various phytohormone treatments (Figure 7). Most previous studies indicate that ABA strongly induces polyamine biosynthetic genes [52]. Although some PA biosynthetic genes in pepper were upregulated by ABA, a greater number showed pronounced expression under IAA and GA3 treatments. It has been reported that GAs positively regulate flowering, trichome formation, and reproductive development [53]. The *CaODC3* gene is significantly induced under GA3 treatment, and also shows significant expression in flowers (Figure 6H), suggesting that PAs and GA may have crosstalk during flower development. However, the relationship of PAs and phytohormones still needs further study. Collectively, the results show that PA biosynthetic genes in pepper respond to a broad spectrum of abiotic stresses and phytohormones, highlighting their important role in enhancing plant stress tolerance.

## 4. Materials and Methods

### 4.1. Plant Growth Conditions and Treatments

The pepper (*Capsicum annuum* L.) cultivar used in this study was ‘Jiaozhongyu’, produced by hybrid breeding of PP1351A × Z121. Pepper seeds were soaked in distilled water in a conical flask and germinated at 28 °C on a shaker. Germinated seeds were sown in 72-well plates filled with a substrate composed of peat, vermiculite, and perlite in a 3:1:1 (*v*/*v*/*v*) ratio. Seedlings were irrigated with Hoagland nutrient solution and grown in an artificial climate chamber under controlled conditions: 25 °C/18 °C (day/night), a 14 h light/10 h dark photoperiod, and a photosynthetic photon flux density (PPFD) of 250 μmol·m^−2^·s^−1^. Seedlings were transplanted into nutrient pots (7 cm diameter) once the third true leaf was fully expanded.

At the six-leaf and one-heart stage, seedlings were subjected to abiotic stress and phytohormone treatments. Abiotic stress treatments included irrigation with 200 mM saline-alkaline mixed solution (NaCl:Na_2_SO_4_:NaHCO_3_:Na_2_CO_3_ = 1:9:9:1, pH 8.90), 200 mM NaCl and 20% polyethylene glycol-6000 (PEG-6000), and foliar spraying with 100 mM hydrogen peroxide (H_2_O_2_), as well as high temperature (42 °C) and low temperature (4 °C) treatments. For phytohormone treatments, leaves were respectively sprayed with 100 μM abscisic acid (ABA), 100 μM indole-3-acetic acid (IAA), 100 μM ethylene (Eth), 100 μM salicylic acid (SA), or 100 μM gibberellin (GA). Leaves were collected at 0 h, 1 h, 6 h, and 12 h after treatment, and the 0 h was used as the control. Samples of roots, stems, leaves, flowers, fruits, and seeds were also collected. All samples were rapidly frozen in liquid nitrogen and stored at −80 °C.

### 4.2. Identification of PA Biosynthesis Genes

Gene and protein sequences of PA biosynthetic genes from *Arabidopsis thaliana* were obtained from The *Arabidopsis* Information Resource (TAIR, http://www.arabidopsis.org, accessed on 1 April 2025). Protein sequences were used as queries for BLASTP searches against the pepper genome with an E-value threshold of <1e^−0^. Sequences from tomato (*Solanum lycopersicum*) were obtained from the Sol Genomics Network (SGN, https://solgenomics.net/, accessed on 1 April 2025). The pepper genome sequence was derived from the telomere-to-telomere gapless genome (PGDB, http://www.pepperbase.site/node/3, accessed on 1 July 2025) [54]. Candidate protein sequences were verified in Pfam (http://pfam.xfam.org/, accessed on 1 April 2025) and NCBI (https://www.ncbi.nlm.nih.gov/, accessed on 1 April 2025).

### 4.3. Gene Structure, Subcellular Localization and Protein Physicochemical Properties Analysis

Coding sequences (CDS), corresponding genomic DNA, and protein sequences of pepper PA biosynthetic genes were extracted from local genomic data using HMMER 3.0 [55]. Exon-intron structures were analyzed using the Gene Structure Display Server (GSDS, http://gsds.cbi.pku.edu.cn, accessed on 3 April 2025) [56]. Protein secondary structures were predicted with the SOPMA method (https://npsa-prabi.ibcp.fr/cgi-bin/npsa_automat.pl?page=/NPSA/npsa_sopma.html, accessed on 6 April 2025) [57]. Subcellular localization was predicted with Euk-mPLoc 2.0 (http://www.csbio.sjtu.edu.cn/bioinf/euk-multi-2/, accessed on 3 April 2025) [58]. Physicochemical properties, including instability index, aliphatic index, isoelectric point, and molecular mass, were calculated using ExPaSy (http://web.expasy.org, accessed on 3 April 2025).

### 4.4. Cis-Element Prediction for PA Biosynthesis Gene Promoters

Promoter sequences (2 kb upstream of the ATG) for each PA biosynthetic gene were extracted from the PGDB and submitted to PlantCARE (http://bioinformatics.psb.ugent.be/webtools/plantcare/html/, accessed on 6 April 2025) for *cis*-element analysis. Visualization of results was performed using TBtools (v1.098745, Guangzhou, China) [59].

### 4.5. Sequence Alignment and Phylogenetic Analysis

Multiple sequence alignments of PA biosynthetic proteins were performed using *ClustalW*. Phylogenetic trees were constructed using the neighbour-joining method in MEGA 7.0 software, with 1000 bootstrap replications, the Poisson model, and pairwise deletion of gaps [49].

### 4.6. Chromosomal Location and Duplication Analysis

Annotations and chromosomal locations of pepper PA biosynthetic genes were obtained from the local genome data according to gene ID. To visualize the location of the genes on the chromosomes using TBtools software (v1.098745, Guangzhou, China). The chromosomal locations of genes in tomato and *Arabidopsis* were identified from SGN website (https://solgenomics.net/, accessed on 7 April 2025) and the TAIR website (http://www.arabidopsis.org, accessed on 1 April 2025), respectively, and a collinearity graph of pepper, tomato and *Arabidopsis* was drawn using TBtools software (v1.098745, Guangzhou, China) [59].

### 4.7. Total RNA Extraction and Gene Expression Analysis

Total RNA was extracted using the PLANTpure Plant RNA Kit (Aidlab, Beijing, China) according to the manufacturer’s instructions. First-strand cDNA was synthesized with HiScript IV All-in-One Ultra RT SuperMix for qPCR (Vazyme, Nanjing, China). Quantitative reverse transcription-polymerase chain reaction (RT-qPCR) was conducted using ChamQ SYBR qPCR Master Mix (Vazyme, Nanjing, China) on an ABI QuantStudio 5 (Applied Biosystems, Waltham, MA, USA). Relative gene expression was calculated using the 2^−ΔΔCt^ method [60], with *CaActin* (Gene ID: CaT2T05g00422) as the internal control. PCR primers were designed using Primer Premier 5 and are listed in Table S1.

## 5. Conclusions

This study identified sixteen PA biosynthesis-related genes in the pepper genome, representing key enzymes responsible for the biosynthesis of Put, Spd, Spm, and Tspm. Gene structure, subcellular localization, phylogenetic relationships, and *cis*-acting regulatory elements were predicted using bioinformatics analyses, and expression profiles were quantitatively examined across different organs and under various abiotic stress and phytohormone treatments. The results demonstrate that pepper PA biosynthetic genes exhibit both evolutionary conservation and diversity, and play important roles in response to abiotic stresses and phytohormones. These findings provide new perspectives for understanding the biological functions of PAs in plant stress adaptation and the screened stress-responsive genes can be applied to molecular breeding for stress-tolerant pepper plants through molecular genetic markers or genetic transformation technology, which offer a foundation for the functional studies of PAs and efforts to improve stress resistance in pepper.

## Figures and Tables

**Table 1:** The polyamine biosynthesis genes in pepper.

Gene ID from PGDB	Name	Position	Coding Sequences (bp)	Intron	Subcellular Localization
CaT2T08g03073	*CaADC1*	Chr08: 184132402~184134576	2175	0	Mitochondrion.
CaT2T10g00793	*CaADC2*	Chr10: 100641424~100645196	1296	4	Mitochondrion.
CaT2T12g01633	*CaAIH*	Chr12: 128776924~128789031	1557	10	Extracell. Mitochondrion.
CaT2T11g00431	*CaCPA*	Chr11: 10439223~10444141	846	7	Cytoplasm.
CaT2T03g01685	*CaODC1*	Chr03: 30721970~30723123	1086	0	Mitochondrion.
CaT2T03g01687	*CaODC2*	Chr03: 30758389~30759591	1203	0	Mitochondrion.
CaT2T04g00108	*CaODC3*	Chr04: 1757160~1758467	1308	0	Mitochondrion.
CaT2T01g03927	*CaSAMDC1*	Chr01: 227578235~227579245	1011	0	Cytoplasm.
CaT2T05g00570	*CaSAMDC2*	Chr05: 19555986~19564951	2469	7	Cytoplasm.
CaT2T06g02607	*CaSAMDC3*	Chr06: 73468151~73469155	1005	0	Cytoplasm. Extracell.
CaT2T11g02366	*CaSAMDC4*	Chr11: 268736228~268737310	1083	0	Cytoplasm. Extracell. Nucleus.
CaT2T11g01658	*CaSPDS*	Chr11: 210985086~210989836	1104	7	Cytoplasm. Nucleus.
CaT2T03g02219	*CaSPMS*	Chr03: 42757075~42764512	1044	9	Cytoplasm.
CaT2T02g00038	*CaACL5-1*	Chr02: 4353693~4360371	1032	9	Cytoplasm. Nucleus.
CaT2T03g04212	*CaACL5-2*	Chr03: 259804076~259808320	756	6	Cytoplasm.
CaT2T08g00347	*CaACL5-3*	Chr08: 74094704~74100057	654	6	Cytoplasm.

**Table 2:** The physicochemical properties of the polyamine biosynthesis proteins in pepper.

Name	Number of Amino Acid (aa)	Molecular Weight (kDa)	Theoretical pI	Instability Index	Aliphatic Index	Grand Average of Hydropathicity
CaADC1	724	78.15	5.08	43.64	88.23	−0.053
CaADC2	431	48.02	6.32	48.50	97.59	−0.058
CaAIH	518	56.44	5.54	30.86	90.62	−0.117
CaCPA	281	31.49	5.78	32.50	78.15	−0.334
CaODC1	361	40.00	7.65	40.41	95.93	−0.060
CaODC2	400	43.89	5.84	41.00	94.57	0.025
CaODC3	435	46.94	5.59	40.71	85.29	0.007
CaSAMDC1	336	37.93	5.40	44.60	79.14	−0.061
CaSAMDC2	822	92.00	7.79	42.51	77.53	−0.360
CaSAMDC3	334	37.51	5.73	44.60	77.90	−0.082
CaSAMDC4	360	39.38	5.28	39.59	82.89	−0.099
CaSPDS	367	40.34	5.09	51.20	87.85	−0.142
CaSPMS	347	38.66	5.17	43.92	87.09	−0.165
CaACL5-1	343	39.08	5.31	34.00	84.14	−0.278
CaACL5-2	251	28.44	5.25	33.27	81.16	−0.414
CaACL5-3	217	24.63	5.40	29.10	77.24	−0.327

## Data Availability

The data from this manuscript has already been submitted in the article or Supplementary Materials. If additional data is required, please contact the authors.

## Supplementary Materials

The following supporting information can be downloaded at: https://www.mdpi.com/article/10.3390/ijms26178208/s1.

## References

[1] Tabor C.W., Tabor H. (1984). Polyamines. Annu. Rev. Biochem..

[2] Takahashi T., Kakehi J. (2010). Polyamines: Ubiquitous polycations with unique roles in growth and stress responses. Ann. Bot..

[3] Xuan M., Gu X., Li J., Huang D., Xue C., He Y. (2023). Polyamines: Their significance for maintaining health and contributing to diseases. Cell Commun. Signal..

[4] Alcázar R., Altabella T., Marco F., Bortolotti C., Reymond M., Koncz C., Carrasco P., Tiburcio A.F. (2010). Polyamines: Molecules with regulatory functions in plant abiotic stress tolerance. Planta.

[5] Fuell C., Elliott K.A., Hanfrey C.C., Franceschetti M., Michael A.J. (2010). Polyamine biosynthetic diversity in plants and algae. Plant Physiol. Biochem..

[6] Chen B.X., Li Y.B., Liu H.P., Kurtenbach R. (2023). Putrescine transformation to other forms of polyamines in filling grain embryos functioned in enhancing the resistance of maize plants to drought stress. Plant Physiol. Biochem..

[7] Shi H., Chan Z. (2014). Improvement of plant abiotic stress tolerance through modulation of the polyamine pathway. J. Integr. Plant Biol..

[8] Takahashi Y. (2024). ACL5 acquired strict thermospermine synthesis activity during the emergence of vascular plants. New Phytol..

[9] Planas-Portell J., Gallart M., Tiburcio A.F., Altabella T. (2013). Copper-containing amine oxidases contribute to terminal polyamine oxidation in peroxisomes and apoplast of Arabidopsis thaliana. BMC Plant Biol..

[10] Tiburcio A.F., Alcázar R. (2018). Potential Applications of Polyamines in Agriculture and Plant Biotechnology. Polyamines: Methods in Molecular Biology.

[11] Richards F.J., Coleman R.G. (1952). Occurrence of Putrescine in Potassium-deficient Barley. Nature.

[12] Pál M., Szalai G., Janda T. (2015). Speculation: Polyamines are important in abiotic stress signaling. Plant Sci..

[13] Gill S.S., Tuteja N. (2010). Polyamines and abiotic stress tolerance in plants. Plant Signal. Behav..

[14] Minocha R., Majumdar R., Minocha S.C. (2014). Polyamines and abiotic stress in plants: A complex relationship. Front. Plant Sci..

[15] Urano K., Yoshiba Y., Nanjo T., Ito T., Yamaguchi-Shinozaki K., Shinozaki K. (2004). Arabidopsis stress-inducible gene for arginine decarboxylase AtADC2 is required for accumulation of putrescine in salt tolerance. Biochem. Biophys. Res. Commun..

[16] Do P.T., Degenkolbe T., Erban A., Heyer A.G., Kopka J., Köhl K.I., Hincha D.K., Zuther E. (2013). Dissecting Rice Polyamine Metabolism under Controlled Long-Term Drought Stress. PLoS ONE.

[17] Li Z.Y., Chen S.Y. (1999). Differential accumulation of the S-adenosyl-methionine decarboxylase transcript in rice seedlings in response to salt and drought stresses. Theor. Appl. Genet..

[18] Zhu X., Li Q., Hu J., Wang M., Li X. (2015). Molecular Cloning and Characterization of Spermine Synthesis Gene Associated with Cold Tolerance in Tea Plant (*Camellia sinensis*). Appl. Biochem. Biotechnol..

[19] Kusbah M.M., Yelenosky G. (1987). Evaluation of Polyamine and Proline Levels during Low Temperature Acclimation of Citrus. Plant Physiol..

[20] Zapata P.J., Serrano M., Pretel M.T., Amorós A., Botella M.Á. (2004). Polyamines and ethylene changes during germination of different plant species under salinity. Plant Sci..

[21] Zhang J., Liang L., Xiao J., Xie Y., Zhu L., Xue X., Xu L., Zhou P., Ran J., Huang Z. (2022). Genome-Wide Identification of Polyamine Oxidase (PAO) Family Genes: Roles of CaPAO2 and CaPAO4 in the Cold Tolerance of Pepper (Capsicum annuum L.). Int. J. Mol. Sci..

[22] Zhang J., Xie M., Yu G., Wang D., Xu Z., Liang L., Xiao J., Xie Y., Tang Y., Sun G. (2023). CaSPDS, a Spermidine Synthase Gene from Pepper (*Capsicum annuum* L.), Plays an Important Role in Response to Cold Stress. Int. J. Mol. Sci..

[23] Xu Z., Sun M., Jiang X., Sun H., Dang X., Cong H., Qiao F. (2018). Glycinebetaine Biosynthesis in Response to Osmotic Stress Depends on Jasmonate Signaling in Watermelon Suspension Cells. Front. Plant Sci..

[24] Yang F., Lu C., Wei Y., Wu J., Ren R., Gao J., Ahmad S., Jin J., Xv Y., Liang G. (2022). Organ-Specific Gene Expression Reveals the Role of the Cymbidium ensifolium-miR396/Growth-Regulating Factors Module in Flower Development of the Orchid Plant Cymbidium ensifolium. Front. Plant Sci..

[25] Napieraj N., Janicka M., Reda M. (2023). Interactions of Polyamines and Phytohormones in Plant Response to Abiotic Stress. Plants.

[26] Ebeed H.T. (2022). Genome-wide analysis of polyamine biosynthesis genes in wheat reveals gene expression specificity and involvement of STRE and MYB-elements in regulating polyamines under drought. BMC Genom..

[27] Alhag A., Song J., Dahro B., Wu H., Khan M., Salih H., Liu J.H. (2021). Genome-wide identification and expression analysis of Polyamine Uptake Transporter gene family in sweet orange (*Citrus sinensis*). Plant Biol..

[28] Liu T., Huang B., Chen L., Xian Z., Song S., Chen R., Hao Y. (2018). Genome-wide identification, phylogenetic analysis, and expression profiling of polyamine synthesis gene family members in tomato. Gene.

[29] Sen S., Ghosh D., Mohapatra S. (2018). Modulation of polyamine biosynthesis in Arabidopsis thaliana by a drought mitigating Pseudomonas putida strain. Plant Physiol. Biochem..

[30] Scott J.D., Pawson T. (2009). Cell Signaling in Space and Time: Where Proteins Come Together and When They’re Apart. Science.

[31] Hillis D.M. (1997). Phylogenetic analysis. Curr. Biol..

[32] Killiny N., Nehela Y. (2020). Citrus Polyamines: Structure, Biosynthesis, and Physiological Functions. Plants.

[33] Chen D., Shao Q., Yin L., Younis A., Zheng B. (2019). Polyamine Function in Plants: Metabolism, Regulation on Development, and Roles in Abiotic Stress Responses. Front. Plant Sci..

[34] Sémon M., Wolfe K.H. (2007). Consequences of genome duplication. Curr. Opin. Genet. Dev..

[35] Moore R.C., Purugganan M.D. (2005). The evolutionary dynamics of plant duplicate genes. Curr. Opin. Plant Biol..

[36] Xie T., Zeng L., Chen X., Rong H., Wu J., Batley J., Jiang J., Wang Y. (2020). Genome-Wide Analysis of the Lateral Organ Boundaries Domain Gene Family in Brassica Napus. Genes.

[37] Hernandez-Garcia C.M., Finer J.J. (2014). Identification and validation of promoters and cis-acting regulatory elements. Plant Sci..

[38] Li Z., Huang Y., Shen Z., Wu M., Huang M., Hong S.B., Xu L., Zang Y. (2024). Advances in functional studies of plant MYC transcription factors. Theor. Appl. Genet..

[39] Dubos C., Stracke R., Grotewold E., Weisshaar B., Martin C., Lepiniec L. (2010). MYB transcription factors in Arabidopsis. Trends Plant Sci..

[40] Sun P., Zhu X., Huang X., Liu J.H. (2014). Overexpression of a stress-responsive MYB transcription factor of Poncirus trifoliata confers enhanced dehydration tolerance and increases polyamine biosynthesis. Plant Physiol. Biochem..

[41] Martínez-Pastor M.T., Marchler G., Schüller C., Marchler-Bauer A., Ruis H., Estruch F. (1996). The Saccharomyces cerevisiae zinc finger proteins Msn2p and Msn4p are required for transcriptional induction through the stress response element (STRE). EMBO J..

[42] Yoshida T., Fujita Y., Sayama H., Kidokoro S., Maruyama K., Mizoi J., Shinozaki K., Yamaguchi-Shinozaki K. (2010). AREB1, AREB2, and ABF3 are master transcription factors that cooperatively regulate ABRE-dependent ABA signaling involved in drought stress tolerance and require ABA for full activation. Plant J..

[43] Song J., Sun P., Kong W., Xie Z., Li C., Liu J.H. (2022). SnRK2.4-mediated phosphorylation of ABF2 regulates ARGININE DECARBOXYLASE expression and putrescine accumulation under drought stress. New Phytol..

[44] Khajuria A., Sharma N., Bhardwaj R., Ohri P. (2018). Emerging Role of Polyamines in Plant Stress Tolerance. Curr. Protein Pept. Sci..

[45] Ohri P., Bhardwaj R., Bali S., Kaur R., Jasrotia S., Khajuria A., Parihar R.D. (2015). The Common Molecular Players in Plant Hormone Crosstalk and Signaling. Curr. Protein Pept. Sci..

[46] Bagni N., Ruiz-Carrasco K., Franceschetti M., Fornalè S., Fornasiero R.B., Tassoni A. (2006). Polyamine metabolism and biosynthetic gene expression in Arabidopsis thaliana under salt stress. Plant Physiol. Biochem..

[47] Alcázar R., Bitrián M., Bartels D., Koncz C., Altabella T., Tiburcio A.F. (2014). Polyamine metabolic canalization in response to drought stress in Arabidopsis and the resurrection plant Craterostigma plantagineum. Plant Signal. Behav..

[48] Cuevas J.C., López-Cobollo R., Alcázar R., Zarza X., Koncz C., Altabella T., Salinas J., Tiburcio A.F., Ferrando A. (2008). Putrescine Is Involved in Arabidopsis Freezing Tolerance and Cold Acclimation by Regulating Abscisic Acid Levels in Response to Low Temperature. Plant Physiol..

[49] Kumar S., Stecher G., Tamura K. (2016). MEGA7: Molecular Evolutionary Genetics Analysis Version 7.0 for Bigger Datasets. Mol. Biol. Evol..

[50] Hao Y.J., Zhang Z., Kitashiba H., Honda C., Ubi B., Kita M., Moriguchi T. (2005). Molecular cloning and functional characterization of two apple S-adenosylmethionine decarboxylase genes and their different expression in fruit development, cell growth and stress responses. Gene.

[51] Zhu M., Chen G., Wu J., Wang J., Wang Y., Guo S., Shu S. (2023). Identification of cucumber S-adenosylmethionine decarboxylase genes and functional analysis of CsSAMDC3 in salt tolerance. Front. Plant Sci..

[52] Yariuchi Y., Okamoto T., Noutoshi Y., Takahashi T. (2021). Responses of Polyamine-Metabolic Genes to Polyamines and Plant Stress Hormones in Arabidopsis Seedlings. Cells.

[53] Banerjee A., Roychoudhury A. (2019). The Regulatory Signaling of Gibberellin Metabolism and Its Crosstalk with Phytohormones in Response to Plant Abiotic Stresses. Plant Signaling Molecules.

[54] Chen W., Wang X., Sun J., Wang X., Zhu Z., Ayhan D.H., Yi S., Yan M., Zhang L., Meng T. (2024). Two telomere-to-telomere gapless genomes reveal insights into Capsicum evolution and capsaicinoid biosynthesis. Nat. Commun..

[55] Potter S.C., Luciani A., Eddy S.R., Park Y., Lopez R., Finn R.D. (2018). HMMER web server: 2018 update. Nucleic Acids Res..

[56] Hu B., Jin J., Guo A.Y., Zhang H., Luo J., Gao G. (2015). GSDS 2.0: An upgraded gene feature visualization server. Bioinformatics.

[57] Geourjon C., Deleage G. (1995). SOPMA: Significant improvements in protein secondary structure prediction by consensus prediction from multiple alignments. Bioinformatics.

[58] Martin D.P., Chou K.C., Shen H.B. (2010). A New Method for Predicting the Subcellular Localization of Eukaryotic Proteins with Both Single and Multiple Sites: Euk-mPLoc 2.0. PLoS ONE.

[59] Chen C., Chen H., Zhang Y., Thomas H.R., Frank M.H., He Y., Xia R. (2020). TBtools: An Integrative Toolkit Developed for Interactive Analyses of Big Biological Data. Mol. Plant.

[60] Livak K.J., Schmittgen T.D. (2001). Analysis of relative gene expression data using real-time quantitative PCR and the 2^−ΔΔCT^ method. Methods.

