# Supplementary material for: Genome-Wide Identification, Phylogenetic Analysis, and Expression Pattern of Polyamine Biosynthesis Gene Family in Pepper
Source: Int J Mol Sci. 2025 Aug 24;26(17):8208. doi: 10.3390/ijms26178208 (PMC12427771; doi:10.3390/ijms26178208)
Supplement: Supplementary file 1 [file ijms-26-08208-s001.zip › Figure S1~8 Alignment analysis of polyamine biosynthetic proteins.pdf]

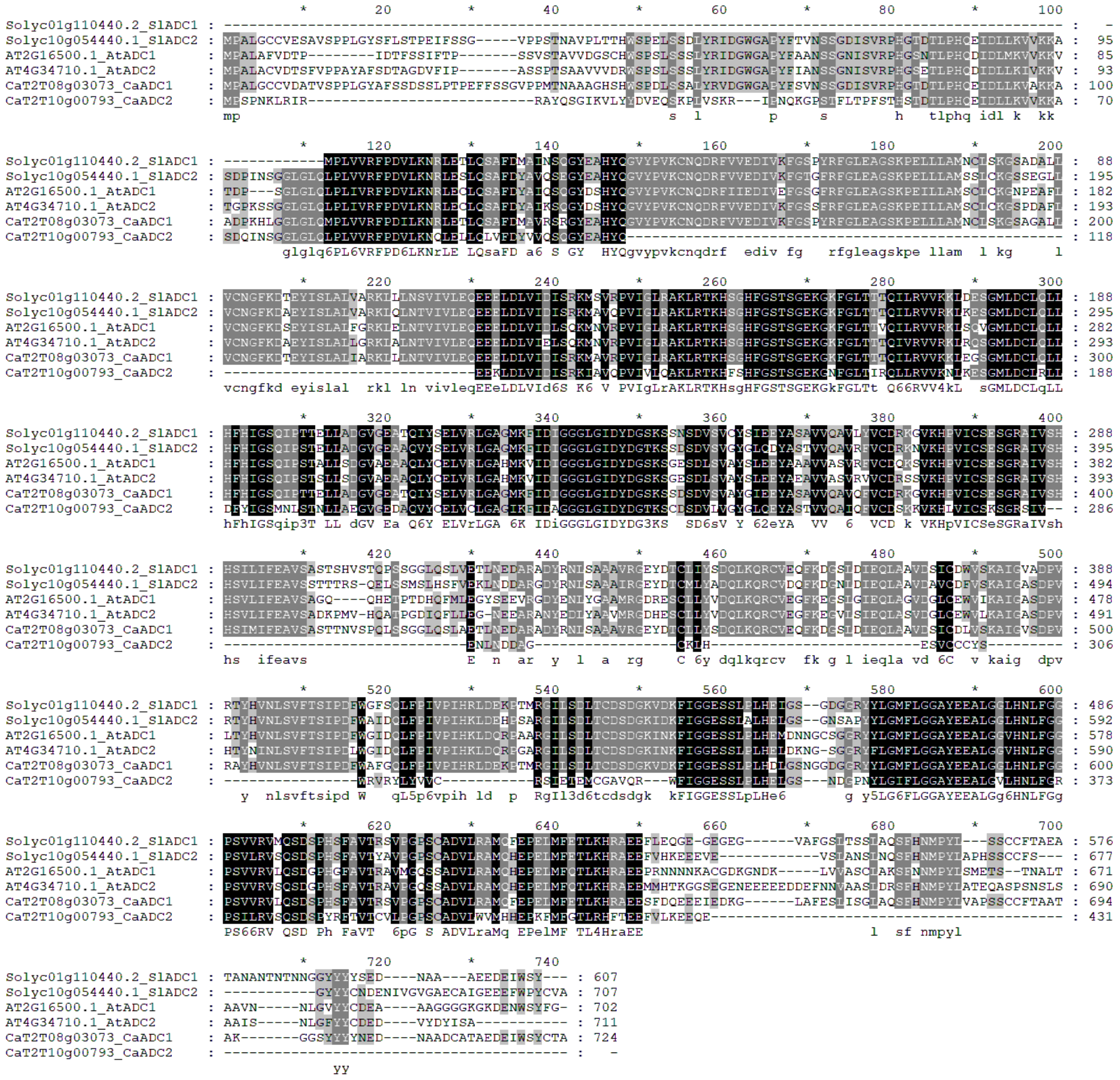

Figure S1. Alignment analysis of arginine decarboxylase (ADC) proteins in pepper (*Capsicum annuun*), tomato (*Solanum lycopersicum*) and Arabidopsis (*Arabidopsis thaliana*).

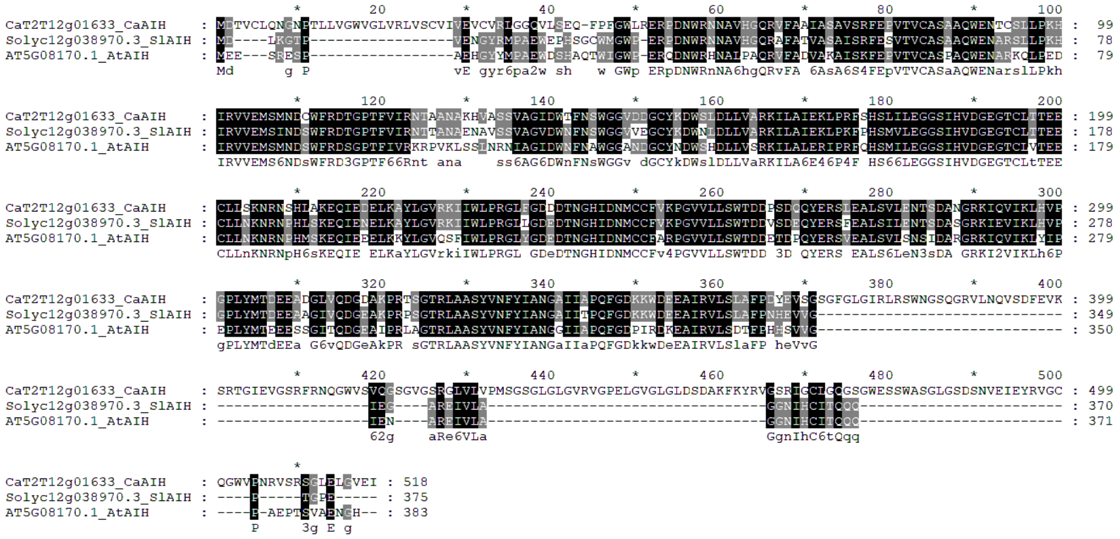

Figure S2. Alignment analysis of agmatine iminohydrolase (AIH) proteins in pepper (*Capsicum annuun*), tomato (*Solanum lycopersicum*) and Arabidopsis (*Arabidopsis thaliana*).

```
                                  *        20         *        40         *        60         *        80         *       100
CaT2T11g00431_CaCPA     : MKPRGK--IGAILRANFLSSP-FSFDLEDKE-----------------LFEGYYFCQAQREDFFHRAKPYQGHPTIVMMQNLAKELGVVIPVSFFEEAN :  79
Solyc11g068540.2_SlCPA  : MAEKNR--LVTVAALQFACTDDVSTNVATAERLVRAAHQKGANIILIQELFEGYYFCQAQKEEFFHRAKPYPGHPTIVRMQNLAKELGVVIPVSFFEEAN :  98
AT2G27450.1_AtCPA       : METEGRRREVVVSSLQFACSDDISTNVAAAERLVREAHAKGANIILIQELFEGYYFCQAQREDFFKRAKPYKNHPTIARMQKLAKELGVVIPVSFFEEAN : 100
                          M   g4   v 6  lqFac3dd St16a aErlvr ah kganiiliqeLFEGYYFCQAQ4EdFFhRAKPY gHPTIvrMQnLAKELGVVIPVSFFEEAN

                                  *       120         *       140         *       160         *       180         *       200
CaT2T11g00431_CaCPA     : NAHYNSVAVIDADGTDLGLYRKSHIPDGPGYQEKFYFNPGDTGFKVFQTKYAKIGVAICWDQWFPEAARALALQGAEVLFYPTAIGSEPQDDGLDSRDHW : 179
Solyc11g068540.2_SlCPA  : NAHYNSVAIIDADGTDLGLYRKSHIPDGPGYQEKYYFNPGDTGFKVFQTKYAKIGVAICWDQWFPEAARAMALQGAEVLFYPTAIGSEPQDDGLDSRDHW : 198
AT2G27450.1_AtCPA       : TAHYNSIAIIDADGTDLGIYRKSHIPDGPGYQEKFYFNPGDTGFKVFQTKFAKIGVAICWDQWFPEAARAMVLQGAEILFYPTAIGSEPQDQGLDSRDHW : 200
                          nAHYNS6A6IDADGTDLG6YRKSHIPDGPGYQEK5YFNPGDTGFKVFQTK5AKIGVAICWDQWFPEAARA6aLQGAE6LFYPTAIGSEPQDdGLDSRDHW

                                  *       220         *       240         *       260         *       280         *       300
CaT2T11g00431_CaCPA     : RRVMQGHAGANVVPLVASNRIGKEIIETEHGNSEITFYGYSFIAGPTGELVAAAGDKEEAVLVAQFDLDKIKSKRHSWGVYRDRRPDLYKVLLTLDGINP : 279
Solyc11g068540.2_SlCPA  : RRVMQGHAGANVVPLVASNRIGKEIIETEHGNSEITFYGYSFIAGPTGELVAAAGDKEEAVLVAQFDLDKIKSKRHGWGVYRDRRPDLYKVLLTLDGSNP : 298
AT2G27450.1_AtCPA       : RRVMQGHAGANVVPLVASNRIGKEIIETEHGPSQITFYGTSFIAGPTGEIVAEADDKSEAVLVAQFDLDMIKSKRQSWGVFRDRRPDLYKVLLTMDG-NL : 299
                          RRVMQGHAGANVVPLVASNRIGKEIIETEHGnS2ITFYGySFIAGPTGE6VAaAgDKeEAVLVAQFDLDkIKSKRhsWGV5RDRRPDLYKVLLT6DG Np

CaT2T11g00431_CaCPA     : AK : 281
Solyc11g068540.2_SlCPA  : VK : 300
AT2G27450.1_AtCPA       : -- :   -
                           k
```

Figure S3. Alignment analysis of N-carbamoylputrescine amidohydrolase (CPA) proteins in pepper (*Capsicum annuun*), tomato (*Solanum lycopersicum*) and Arabidopsis (*Arabidopsis thaliana*).

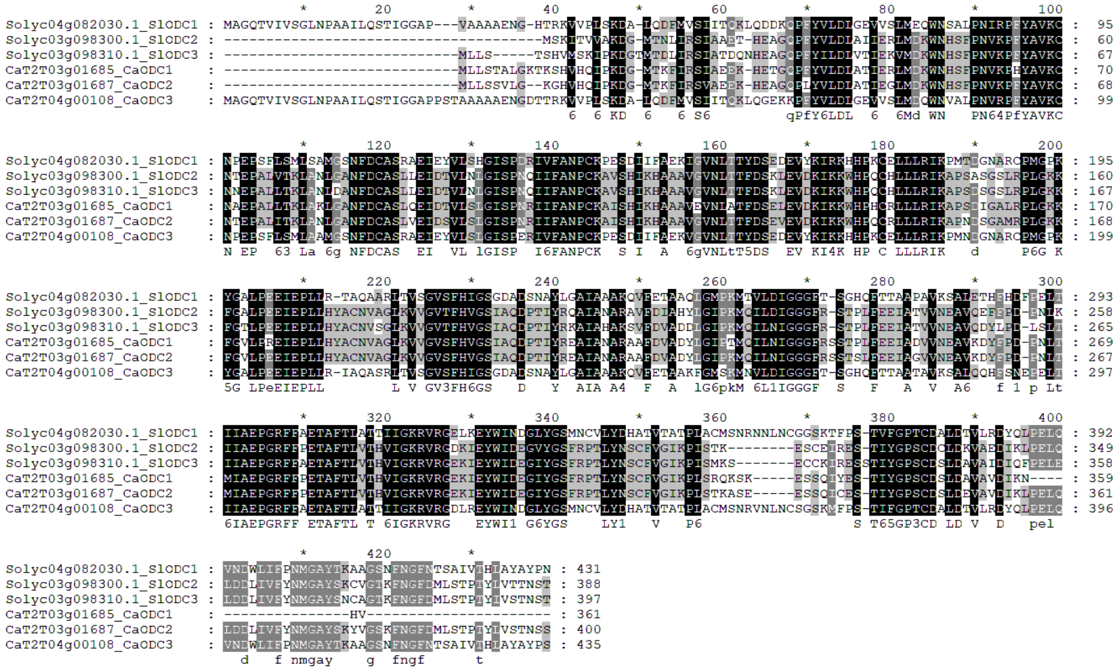

Figure S4. Alignment analysis of ornithine decarboxylase (ODC) proteins in pepper (*Capsicum annuun*) and tomato (*Solanum lycopersicum*).

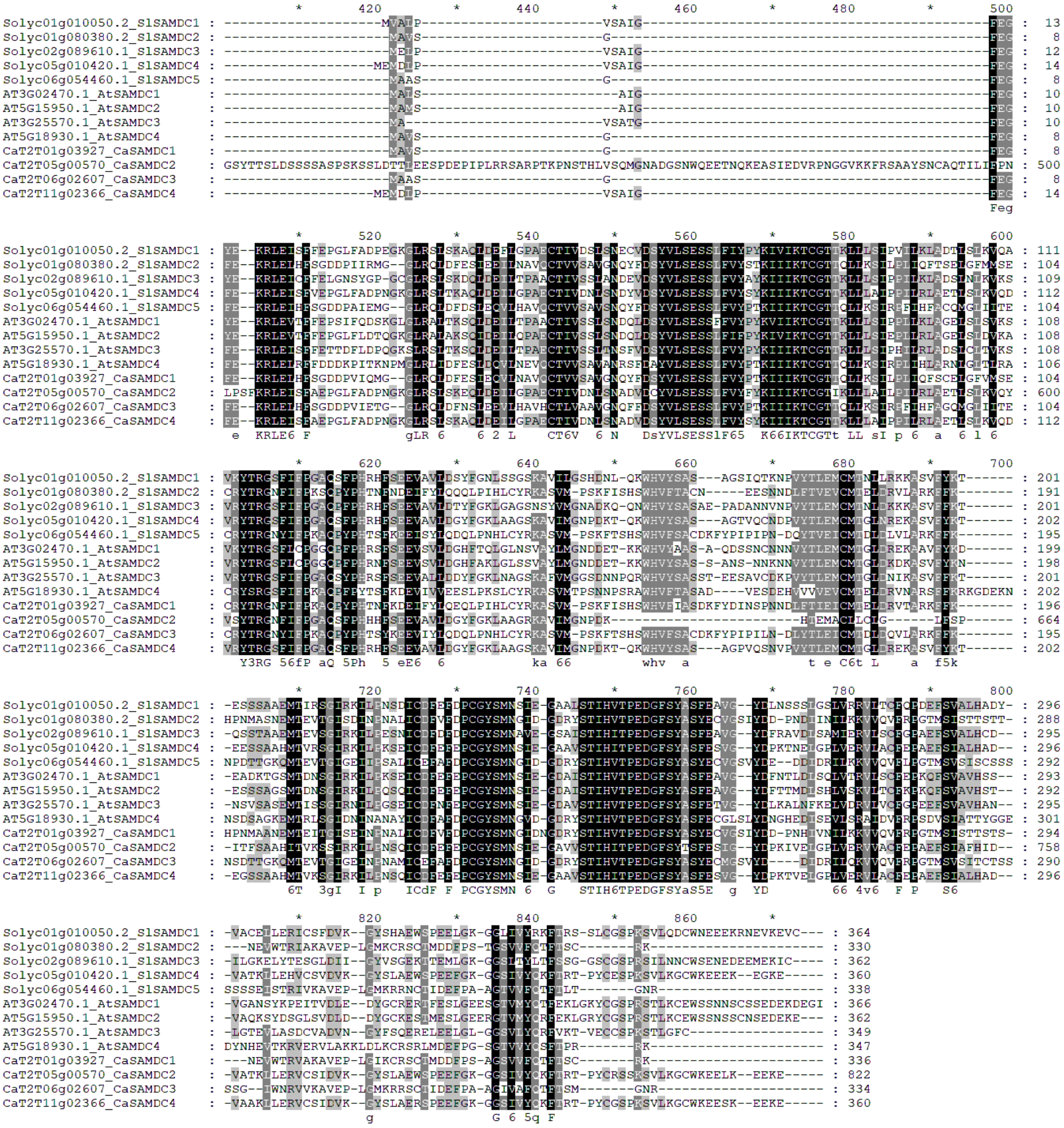

Figure S5. Alignment analysis of S-adenosylmethionine decarboxylase (SAMDC) proteins in pepper (*Capsicum annuun*), tomato (*Solanum lycopersicum*) and Arabidopsis (*Arabidopsis thaliana*).

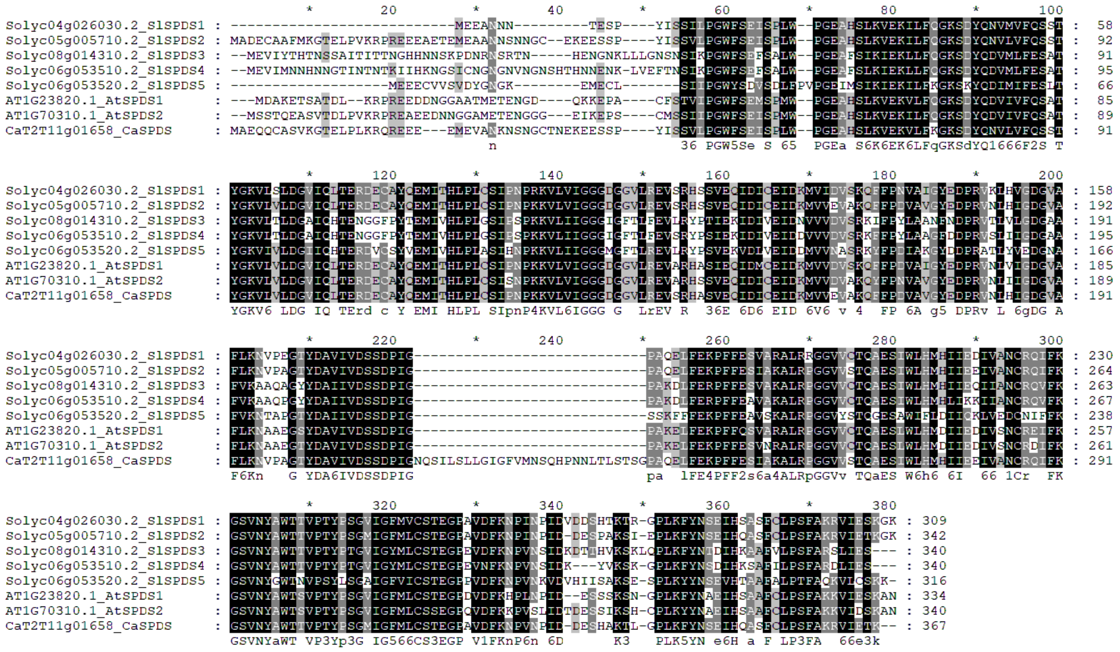

Figure S6. Alignment analysis of spermidine synthase (SPDS) proteins in pepper (*Capsicum annuun*), tomato (*Solanum lycopersicum*) and Arabidopsis (*Arabidopsis thaliana*).

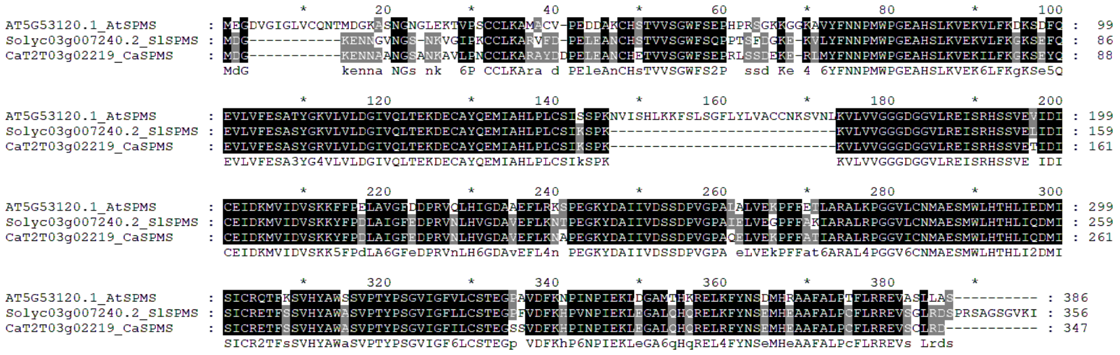

Figure S7. Alignment analysis of spermine synthase (SPMS) proteins in pepper (*Capsicum annuun*), tomato (*Solanum lycopersicum*) and Arabidopsis (*Arabidopsis thaliana*).

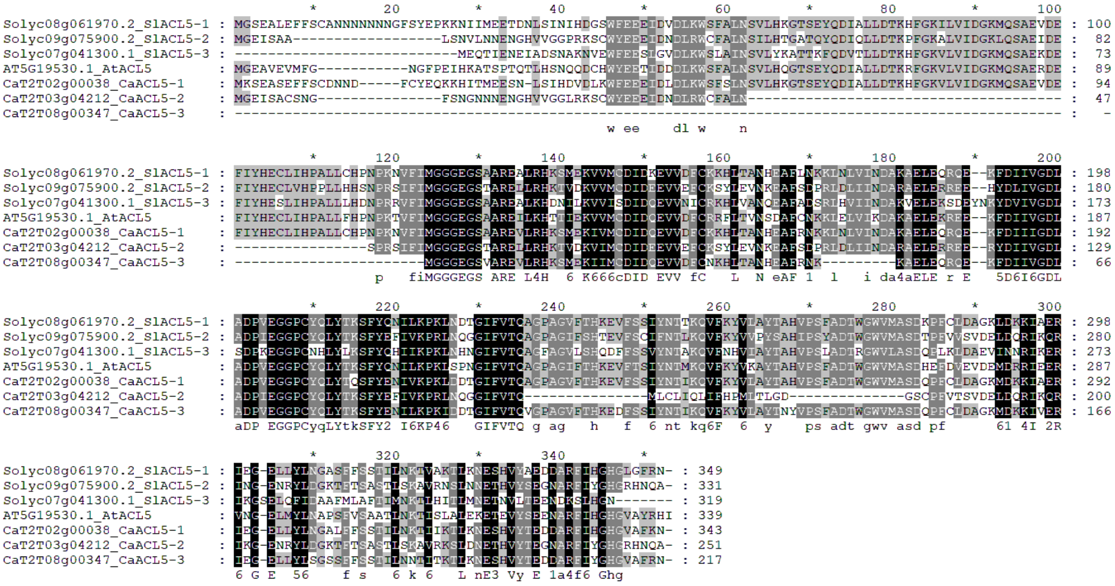

Figure S8. Alignment analysis of ACAULIS5 (ACL5) proteins in pepper (*Capsicum annuun*), tomato (*Solanum lycopersicum*) and Arabidopsis (*Arabidopsis thaliana*).
